# The role and mechanisms of multiple immunoregulatory cells in pulmonary tuberculosis

**DOI:** 10.3389/fimmu.2026.1841581

**Published:** 2026-06-02

**Authors:** Hao Liu, Ping Li, Sujuan He, Haoran Yang, Ting Yuan, Xiaojin He, Yingzheng Tan

**Affiliations:** 1 Department of Infectious Diseases, The Second Hospital of Zhuzhou City, Zhuzhou, Hunan, China; 2 Zhuzhou Clinical College, Jishou University, Zhuzhou, Hunan, China; 3 Department of Infectious Disease, Zhuzhou Hospital Affiliated to Xiangya School of Medicine, Central South University, Zhuzhou, Hunan, China

**Keywords:** immunosuppressive mechanisms, pulmonary tuberculosis, regulatory B cells, regulatory T cells, therapeutic targets

## Abstract

Pulmonary tuberculosis (PTB) is a complex respiratory illness and a leading cause of mortality from infectious diseases globally. The regulation of the host immune response is integral to the chronic infection process of PTB. In this process, the excessive activation of the host’s negative immune regulatory mechanisms serves as the core pathological basis driving infection chronicity and facilitating pathogen immune evasion. Many reviews highlight successful therapeutic approaches aimed at regulatory immune cells. Understanding the roles of various regulatory immune cells in PTB is of paramount importance. This paper examines key regulatory immune cells involved in the PTB infection process, such as regulatory T cells (Tregs), regulatory B cells (Bregs), myeloid-derived suppressor cells (MDSCs), and macrophages. Furthermore, the study delves into the mechanisms by which regulatory immune cells induce immunosuppressive functions. In summary, gaining a deep understanding of the immunosuppressive network in PTB will provide a theoretical basis for developing more effective new immunotherapy regimens.

## Introduction

Tuberculosis (TB) is a widespread chronic infectious disease predominantly caused by the Mycobacterium tuberculosis (Mtb) complex and transmitted via airborne particles (1, 2). While TB primarily affects the pulmonary system, it has the potential to involve nearly any organ in the body (1). The Bacillus Calmette-Guerin (BCG) vaccine has played a crucial role in reducing TB incidence in conjunction with societal advancements (3). Despite the notable decline in TB cases, the emergence of multi-drug-resistant (MDR-TB) and extensively drug-resistant TB (XDR-TB) strains poses significant challenges to clinical management (4).

Cellular immunity, predominantly mediated by Th1 cells that produce interferon-gamma (IFN-γ) to activate macrophages, constitutes the principal mechanism of host resistance to TB, aiding in the elimination or suppression of intracellular Mtb (5). Nonetheless, the efficacy of this immune response necessitates sophisticated negative regulatory mechanisms to avert excessive inflammatory reactions, thereby reducing immune-mediated inflammatory damage to the host (6). Within this intricate immune regulatory framework, various regulatory cells are involved, primarily including Tregs, Bregs, MDSCs, and M2 macrophages (7, 8). Evidence suggests that tertiary lymphoid structures (TLS) enhance immune cell interactions, thereby playing a crucial role in TB immune regulation (9). These regulatory cells are essential for maintaining cellular homeostasis and preventing autoimmune damage (7, 8).

In TB patients, regulatory cells, which are expected to function as a “braking mechanism, “ exhibit a dual role (10, 11). On one hand, these cells are capable of finely modulating the immune response to effectively eliminate Mtb, thereby reducing host immune-mediated inflammatory damage and preserving lung tissue function (11). On the other hand, sites of Mtb infection can result in a substantial accumulation of regulatory cells. This excessive immunosuppression may compromise the host’s protective immunity, allowing the bacteria to evade the immune response, which in turn facilitates the prolonged survival of Mtb within the host and accelerates disease progression (10). As research into the immune mechanisms of TB continues to advance, immunotherapy has emerged as a pivotal focus within TB research (12). When combined with standard anti-tuberculosis treatments, immunotherapy holds promise for enhancing treatment outcomes, particularly in patients with MDR-TB and XDR-TB (12, 13).

This review systematically explores the roles and molecular mechanisms of various regulatory cells in the development and progression of PTB. We anticipate that this will provide guidance and a theoretical foundation for the development of novel immunotherapy strategies.

## Tregs

Tregs represent a subset of lymphocytes that play a critical role in the negative regulation of the immune response within the immune microenvironment of pulmonary tuberculosis. Among these, CD4+CD25+FOXP3+ Tregs are the predominant subpopulation (14). The transcription factor FoxP3 is essential for the development and functional capacity of Tregs, with its expression level being directly associated with the immunosuppressive efficacy of these cells (15). Tregs are categorized based on their developmental origins into thymically derived Tregs (tTregs), peripheral Tregs (pTregs) that originate from immature CD4+ T cells in the periphery under the influence of cytokines such as TGF-β and IL-2, and induced Tregs (iTregs) that are generated through *in vitro* manipulation (15, 16). In the context of Mtb infection, Tregs maintain immune homeostasis by suppressing excessive inflammatory responses, thereby preventing tissue damage and modulating the progression of PTB (17).

**Figure 1:**
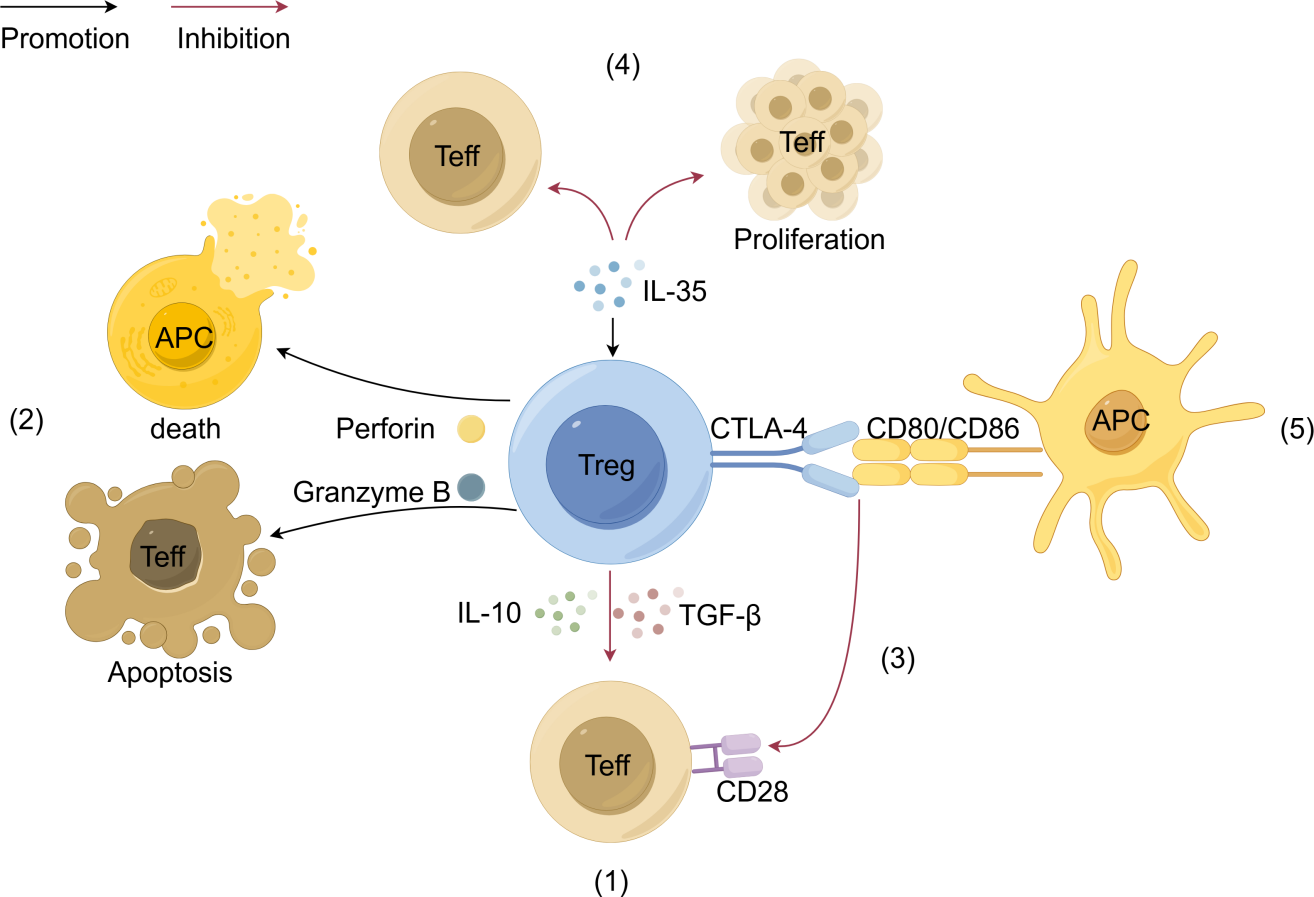
The immunosuppressive mechanisms of Tregs. (1) Tregs suppress the function of effector T cells (Teff) by releasing immunosuppressive cytokines, including IL-10 and TGF-β. (2) They exert immunoregulatory effects by lysing APCs through PRF and GZB-dependent mechanisms. (3) They indirectly inhibit Teff expression by competitively blocking CD28 co-stimulation. (4) IL-35 is involved in regulating Treg function and can suppress Teff proliferation and function. (5) Tregs regulate APC expression by influencing the binding of CTLA-4 to CD80/CD86.

Research indicates that patients with PTB exhibit a significantly higher proportion of Tregs in their peripheral blood compared to individuals with latent tuberculosis infection (LTBI) or healthy controls. This finding suggests a potential association between Treg expansion and the progression of PTB (18, 19). Furthermore, the study identified a markedly increased proportion of CD4+CD25+FOXP3+ Tregs in the peripheral blood of patients with MDR-TB relative to healthy individuals, suggesting a possible link between Tregs and TB drug resistance (20). In terms of the mechanisms by which Tregs exert their immunosuppressive functions, several pathways have been identified. It has been demonstrated that IFN-γ is essential for macrophage activation to eradicate intracellular Mtb (5). Tregs can suppress effector T cell function by secreting IL-10 and TGF-β, thereby disrupting IFN-γ production (21). Interleukin-35 (IL-35) is a critical immunosuppressive cytokine involved in the regulation of Treg function (22). IL-35 directly inhibits the proliferation and function of effector T cells while promoting the generation of iTregs, thereby facilitating infection tolerance (23). Secondly, iTreg mediates its immunoregulatory effects by lysing antigen-presenting cells (APCs) through mechanisms dependent on perforin (PRF) and granzyme B (GZB) (24). Furthermore, Tregs exert indirect inhibitory effects by engaging cytotoxic T-lymphocyte-associated protein 4 (CTLA-4) expressed on T cells with CD80/CD86, thereby competitively inhibiting CD28 co-stimulation and indirectly suppressing the activation of effector T cells (25, 26). These mechanisms enable Tregs to impose negative regulatory effects on the immune response, preventing excessive immune damage during the progression of PTB while simultaneously facilitating the survival and proliferation of Mtb. Research has demonstrated that the transcription factor YY1 in Tregs suppresses FOXP3 expression and diminishes Treg inhibitory function by obstructing the binding of Smad3/4 to the FOXP3 promoter site and interfering with FOXP3-dependent target gene expression (27). YY1 may influence the differentiation and function of Treg cells by downregulating FOXP3 expression and activity, thereby contributing to the maintenance of immune homeostasis. In murine models, a decrease in Tregls has been correlated with heightened pulmonary inflammation and an elevated bacterial burden, indicating that Tregs are integral to modulating inflammation and preserving immune homeostasis (28). Research indicates that Tregs may proliferate during the initial stages of infection to mitigate excessive immune reactions, yet may be selectively depleted in later stages to avert immunosuppression against the pathogen (29). This dynamic change further underscores the complex role of Tregs in the course of infection. Following Mtb infection, Treg cells are involved in the formation of granulomas. Initially, pulmonary granulomas serve to contain the spread of the bacteria; however, severe inflammation during later stages of infection can lead to necrosis of lung tissue and cavity formation, facilitating bacterial dissemination (30, 31). Tregs are essential for preserving the structure of granulomas and maintaining immune balance, but they might weaken antimicrobial immunity (31). On a positive note, moderately activated Treg cells can effectively modulate excessive inflammation induced by Mtb infection to a certain degree (6). Conversely, an increased presence of Treg cells within granulomas may be associated with Mtb’s ability to evade the immune system, potentially playing a significant role in latent tuberculosis infection and recurrence (30). The immunosuppressive mechanisms of Treg cells are illustrated in **Figure 1**.

Considering the essential function of Tregs in regulating the immune response to TB, focusing on these cells has become a promising approach for TB immunotherapy (25). Numerous studies have demonstrated that immune checkpoint molecules, including programmed death receptor-1 (PD-1), cytotoxic T-lymphocyte-associated protein 4 (CTLA-4), lymphocyte activation gene-3 (LAG-3), T cell immunoglobulin and ITIM domain (TIGIT), and T cell immunoglobulin and mucin domain-3 (TIM-3), are integral to immune regulation during TB. These molecules thus represent potential therapeutic targets for TB treatment (25). Specifically, research has identified CTLA-4 as a key regulator of the immune function of FOXP3+ Tregs. Modulating CTLA-4 activity may alter Treg function to enhance anti-tuberculosis immunity, thereby providing a foundation for innovative immunotherapeutic strategies against TB (25, 32). Additionally, evidence suggests that PD-1 and its ligands can inhibit both innate and adaptive immune responses in TB patients (33). However, studies have also shown that inflammation is exacerbated in PD-1-deficient mice infected with Mtb, indicating that PD-1 may confer a protective effect against TB in murine models (34). Consequently, monitoring PD-1 expression may facilitate the evaluation of the host’s protective immune response during tuberculosis treatment. It is important to exercise caution when employing immune checkpoint inhibitors in therapies targeting Tregs (33). TIM-3, a type I transmembrane protein present on various immune cells, is upregulated by Mtb in patients with PTB, thereby modulating the immune response through its effects on Tregs and effector T cells. Research indicates that blocking TIM-3 and its ligand, Galectin-9 (Gal9), may decrease the release of inflammatory cytokines like IFN-γ and TNF-α in T cells (35, 36). Thus, regulating TIM-3 expression may control the inflammatory response in PTB, thereby enhancing Mtb clearance and improving cellular immune function (35).

Follicular regulatory T (Tfr) cells are a unique group of Tregs that influence the germinal center response. By restraining the excessive growth of follicular helper T (Tfh) cells and germinal center B cells, Tfr cells primarily support the selection of high-affinity B cells (37). Tfr cells are integral not only in the suppression of autoimmune responses but also in modulating immune responses post-vaccination. Empirical evidence suggests that Tfr cells regulate somatic hypermutation and clonal diversity within the germinal center response by mitigating clonal competition following SARS-CoV-2 vaccination (38). In the setting of an influenza virus infection, Tfr cells improve antigen-specific germinal center B cell responses and are key to establishing enduring humoral immune memory (39). Furthermore, clinical investigations have shown that traditional Chinese medicine formulas, when used in conjunction with conventional anti-tuberculosis medications, hold promise for the modulation of Tregs (40). In a study involving patients with PTB, it was observed that both the cohort receiving a combined therapy of the traditional Chinese medicine compound Baihe Gujin decoction (BGD) and conventional anti-tuberculosis drugs, and the cohort receiving only standard anti-tuberculosis therapy, experienced a significant reduction in the proportion of CD4+CD25+FOXP3+ Tregs in peripheral blood over the course of treatment. However, in bronchoalveolar lavage fluid (BALF), the reduction in CD4+CD25+FOXP3+ Tregs was significantly lower in the combination therapy group compared to the conventional anti-tuberculosis therapy group (40). Additionally, the integration of traditional Chinese medicine formulas with PTB treatment was observed to significantly alleviate clinical symptoms and reduce adverse reactions associated with standard anti-tuberculosis medications (40, 41). Nonetheless, this study is subject to certain limitations, primarily due to its small sample size. Furthermore, the mechanisms underlying the differential alterations in Tregs between local and peripheral blood remain inadequately understood. It remains to be elucidated whether components of traditional Chinese medicine exert a direct regulatory effect on Treg function or whether they indirectly influence it by ameliorating the overall inflammatory status. Despite these uncertainties, the initial clinical observations offer preliminary insights into the potential for restoring immune balance in tuberculosis patients. However, the precise mechanisms and clinical implications require validation through studies with larger sample sizes. Furthermore, advancements in cell engineering technologies have highlighted the significant potential of chimeric antigen receptor (CAR)-Treg therapy in the contexts of tumor treatment and transplantation. Emerging cell therapies may facilitate the direct *in vivo* generation of engineered Tregs by utilizing metabolic modulators and innovative synthetic receptors (42, 43). Although the investigation of this approach in TB is still in its early stages, it is theoretically conceivable that targeted delivery of engineered Tregs within the body could be realized in the future (42). It is important to acknowledge that this concept remains theoretical at present, and the application of CAR-Tregs in tuberculosis is an unexplored domain. Any endeavor towards clinical translation must prioritize the validation of safety and efficacy through rigorous testing in tuberculosis animal models. Even with successful outcomes, the journey to practical implementation is likely to be protracted. The BCG vaccine was the first developed to prevent tuberculosis, aiming to elicit antigen-specific T cell responses upon Mtb exposure, thereby establishing a durable memory response for extended protection against infection (44). Research has demonstrated that the Akt inhibitor MK-2206 enhances BCG-mediated immune responses by activating FOXO3, which suppresses IL-10 secretion and promotes host cell apoptosis (45). Subsequent studies in murine models infected with Mtb have shown that appropriate caloric restriction (CR) significantly reduces the recruitment of CD3+CD4+CD8+FOXP3+ cells to the lungs and effectively controls bacterial replication within the pulmonary system. This finding suggests a promising therapeutic strategy for PTB treatment (46). Mtb modulates Tregs function through various mechanisms to evade immune clearance. A comprehensive understanding of these mechanisms could identify several potential targets for the development of novel therapeutic strategies.

## Bregs

Bregs constitute a specialized subset of B cells characterized by their immunosuppressive capabilities, primarily modulating immune responses via the secretion of inhibitory cytokines such as IL-10, IL-35, and TGF-β, as well as through cell membrane-associated molecules (47). Phenotypically, human Bregs encompass various subsets, including the transitional CD19CD24+hiCD38hi type, the memory CD24hiCD27+ type, and IgA-producing CD138+ plasma cells (47, 48). The CD19+CD5+CD1d+ Bregs are regarded as a core phenotype within human peripheral blood, producing IL-10 and TGF-β1, and thereby exerting regulatory effects during acute inflammation and immune responses (49). The CD24+hiCD38hi and CD24hiCD27+ Bregs are the primary phenotypes responsible for IL-10 production, demonstrating distinct efficiencies in suppressing CD4+ T cell proliferation and IFN-γ/IL-17 expression (50). These Breg subpopulations differentiate in response to various microenvironmental signals and execute their immunosuppressive functions through multiple molecular pathways (47, 48).

**Figure 2:**
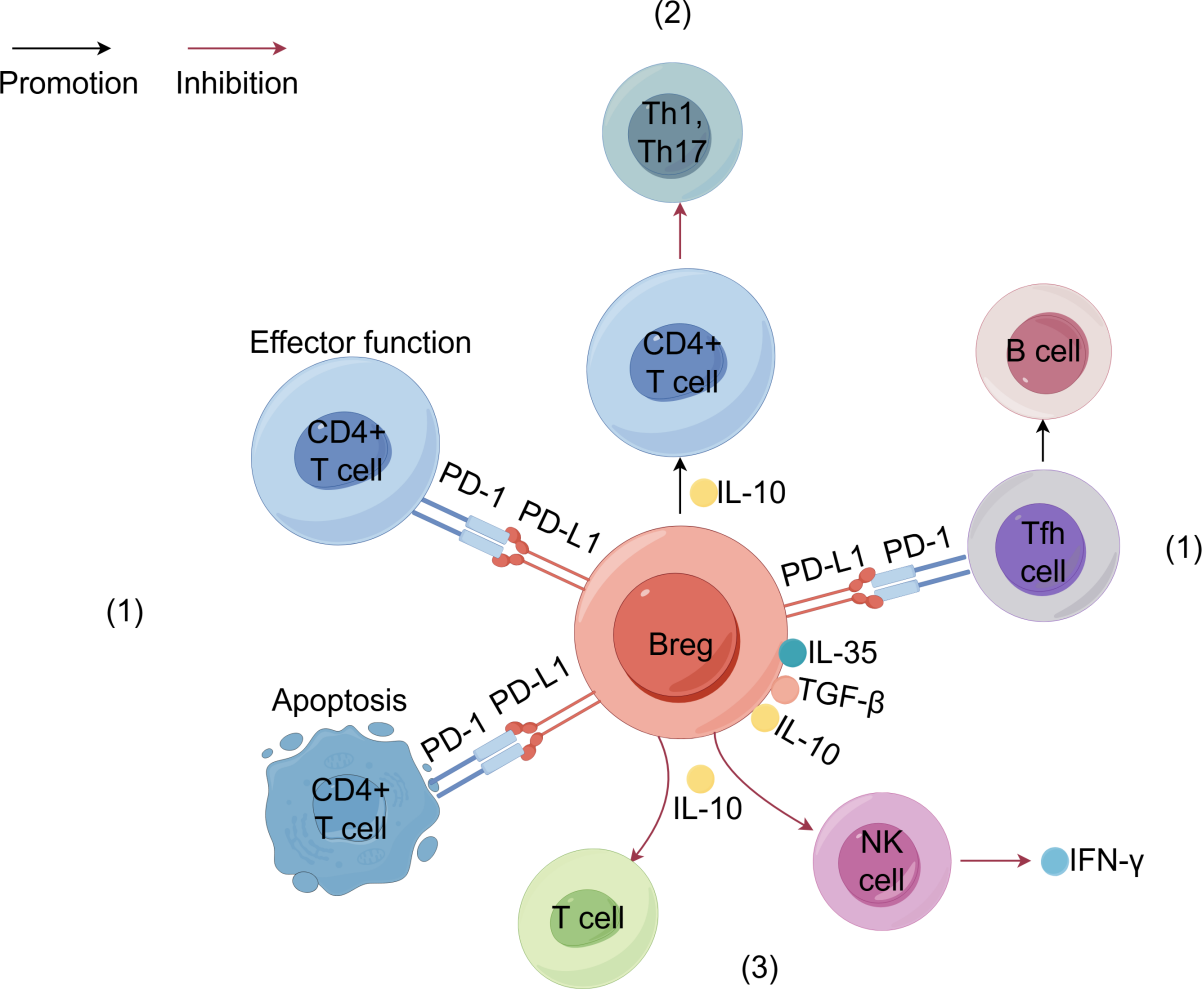
Immunosuppressive mechanisms of bregs. (1) Bregs induce apoptosis and suppress CD4^+^ T cell function by expressing PD-L1 that binds to PD-1 on T cells, while also regulating follicular helper T cell activity. (2) Bregs inhibit CD4^+^ T cell differentiation and function by producing IL-10. (3) Bregs also suppress IFN-γ production by NK cells through IL-10 secretion.

During Mtb infection, Bregs exhibit a paradoxical dual role, encompassing both protective and pathogenic functions. They mitigate tissue damage by suppressing excessive inflammatory responses; however, their potent immunosuppressive capabilities also compromise the host’s effective antimicrobial defense (51). Bregs achieve this by inhibiting IFN-γ production through the secretion of multiple inhibitory cytokines, thereby limiting macrophage activation and bactericidal capacity (52). IL-10, a key immunosuppressive cytokine, has been shown to inhibit IFN-γ production by natural killer (NK) cells in TB patients and significantly reduce T-cell activity (53). Additionally, certain Bregs express surface molecules such as programmed death-ligand 1 (PD-L1), which interact with corresponding receptors on T cells, inducing apoptosis in effector T cells and suppressing their function (54). Studies have demonstrated a significant increase in the frequency of CD19+CD1d+CD5+ Bregs in the peripheral blood of TB patients. These cells notably suppress the activation of Th17 cells while having a minimal effect on Th1 cell activation (55). This evidence suggests that CD19+CD1d+CD5+ Bregs may modulate CD4+ T cell responses by inhibiting Th17 cell activation, thereby affecting the clinical outcomes of tuberculosis. Previous studies have identified CD19+CD5+CD1d+ Bregs using flow cytometry and have demonstrated that these cells produce anti-inflammatory cytokines, such as IL-10 and TGF-β1 (49). These findings imply that during Mtb infection, CD19+CD5+CD1d+ Bregs regulate the immune response by suppressing Th17 cell activation and secreting anti-inflammatory cytokines, thus preventing excessive inflammatory damage. IL-10 producing B cells, known as B10 cells, represent a subset of Bregs. The Mtb-specific surface molecule, mannosyl-capped lipoarabinomannan (ManLAM), interacts with B10 cells, inducing elevated IL-10 expression and subsequently suppressing CD4+ Th1 immune responses. This suggests that Breg activation is intricately linked to specific Mtb surface molecules (56). Given that B10 cells are predominantly located within the CD24hiCD27+ Bregs subpopulation, it is plausible that this CD24hiCD27+ Bregs subset may also be activated through recognition of ManLAM (56). Research indicates that CD24hiCD27+ Bregs decrease TNF-α production in a range of chronic inflammatory and autoimmune conditions without involving IL-10 (57). In the context of chronic MTB infection, CD24hiCD27+ Bregs may similarly modulate inflammatory cytokine production through this pathway. A prospective comparative study revealed that the proportion of CD24+hiCD38hi Bregs is elevated in TB patients compared to healthy individuals (58). These CD24+hiCD38hi Bregs predominantly inhibit CD4+ T cell proliferation by modulating IL-10 production and suppress the secretion of IFN-γ and TNF-α (59). This regulatory function may protect tissues from excessive inflammatory damage during MTB infections; however, excessive activity can compromise bacterial clearance. In a murine model of TB, studies have shown that after B-cell-derived IL-10 was specifically knocked out, the murine exhibited significantly enhanced resistance to MTB, with delayed onset of clinical symptoms and prolonged survival (60). Furthermore, this protective effect was found to be more pronounced in male mice than in female mice, accompanied by male-specific changes in the immune profile (60). These findings may offer a novel molecular perspective for elucidating the gender differences observed in the epidemiology of TB. Given that Th1 cells are crucial effector cells in macrophage activation, their functional suppression results in impaired macrophage activation and diminished bacterial clearance capacity (61). Additionally, in a murine model of TB, Bregs have been observed to produce elevated levels of the cytokine IL-35, which works in concert with IL-10 to enhance Treg upregulation and suppress Th1/Th17 cell activity (62). Tfh cells are key in the activation of B cells and their development into antibody-producing cells, whereas Bregs influence humoral immunity by modulating Tfh function (63). Furthermore, Bregs have the capacity to suppress the production of TNF-α by macrophages, a critical regulator in TB infection control. This suppression may compromise the macrophages’ ability to eliminate Mtb (57). Th17 cells contribute to neutrophil recruitment and granuloma formation through the secretion of IL-17 and other inflammatory mediators, thereby playing a crucial role in the host defense against Mtb (64). Bregs may prevent T cells from differentiating into Th17 cells, which could impact granuloma function and speed up PTB progression (59). Notably, one study suggests that anti-tuberculosis therapy enhances the IL-22 response to specific antigens by decreasing the frequency of CD19+CD5+CD1d+ Bregs (65). This finding implies that Bregs may modulate the immune response to tuberculosis by suppressing IL-22 production (65). **Figure 2** illustrates the mechanisms by which Bregs suppress the immune system.

Recognizing the pivotal immunoregulatory role of Bregs in the progression of PTB, the strategic targeting of Bregs has emerged as a novel focus within tuberculosis immunotherapy. The fundamental principle involves selectively modulating the immunosuppressive functions of Bregs rather than their complete ablation, thereby achieving an equilibrium between immunosuppression and immune protection. Empirical studies suggest that specific cytokine combinations can induce the differentiation of bone marrow B cells into Bregs and promote their proliferation, consequently mitigating immunopathological damage in models of autoimmune diseases. These findings offer valuable insights for PTB therapeutic approaches (66). Crucially, the modulation of Bregs’ function can be accomplished by altering the cytokines they secrete or by inhibiting associated signaling pathways (67, 68). For instance, the immunosuppressive effects of IL-10 secreted by Bregs can be attenuated through the application of neutralizing antibodies, potentially diminishing their suppression of protective immunity and thereby enhancing the anti-tuberculosis immune response (67). Additionally, employing specific antibodies to block PD-L1 on the surface of Bregs may relieve the inhibition on T cells, thus restoring the anti-tuberculosis activity of effector T cells (68). Tryptophan metabolism represents a fundamental mechanism underpinning the immunosuppressive function of Bregs. Modulation of this metabolic pathway may alter their functional state, presenting a novel strategy for PTB immunotherapy (69, 70). B cells located in the splenic marginal zone play a crucial role in controlling Mtb infection by influencing cytokine production and cell-mediated immune responses (71). During tuberculosis infection, these B cells accumulate and exhibit an activated, memory-like phenotype that is distinct from conventional B cells. Emphasizing this regulatory role could be advantageous in the development of a tuberculosis vaccine (71). Furthermore, research suggests that vitamin D regulates the differentiation and function of various cell types, including Bregs, through its interaction with nuclear receptors. The potential use of vitamin D or its analogues to target Bregs may offer an adjunctive therapeutic strategy for PTB (72). TLS are aggregates formed by immune cells within inflamed tissues that facilitate the interaction and activation of local immune cells (9). Research suggests that B cells within the TLS may impact disease progression and patient prognosis in non-small cell lung cancer (NSCLC) by modulating the activation status and differentiation pathways of CD4+ T cells. This implies that B cells, influenced by regulatory T cells, might play a comparable role in PTB (73). Targeting the interactions between B cells and CD4+ T cells in TLS could modify the pulmonary immune microenvironment, potentially limiting the accumulation and function of Bregs (9, 73). As the mechanisms of Bregs in TB are further elucidated, precision immunotherapies targeting Bregs offer promising potential as a significant complement to traditional drug treatments, providing new strategies for TB management.

## MDSCs

MDSCs represent a heterogeneous population of immature myeloid cells that are present in minimal quantities in healthy individuals. However, their numbers increase markedly under pathological conditions such as chronic infections and tumorigenesis, where they exert a profound influence on immune responses (74, 75). Based on phenotypic features, MDSCs are chiefly categorized into two subtypes: polymorphonuclear MDSCs (PMN-MDSCs) and monocytic MDSCs (M-MDSCs) (76). In humans, M-MDSCs are characterized by the expression markers CD11b+CD14+CD33+HLA-DRlow/neg, whereas PMN-MDSCs are identified by CD11b+CD15+HLA-DRlowCD66b+ (74). Importantly, PMN-MDSCs express the lectin-type oxidized low-density lipoprotein receptor 1 (LOX-1), and the level of LOX-1 expression on PMN-MDSCs is closely linked to the immunosuppressive capabilities of MDSCs (77).

**Figure 3:**
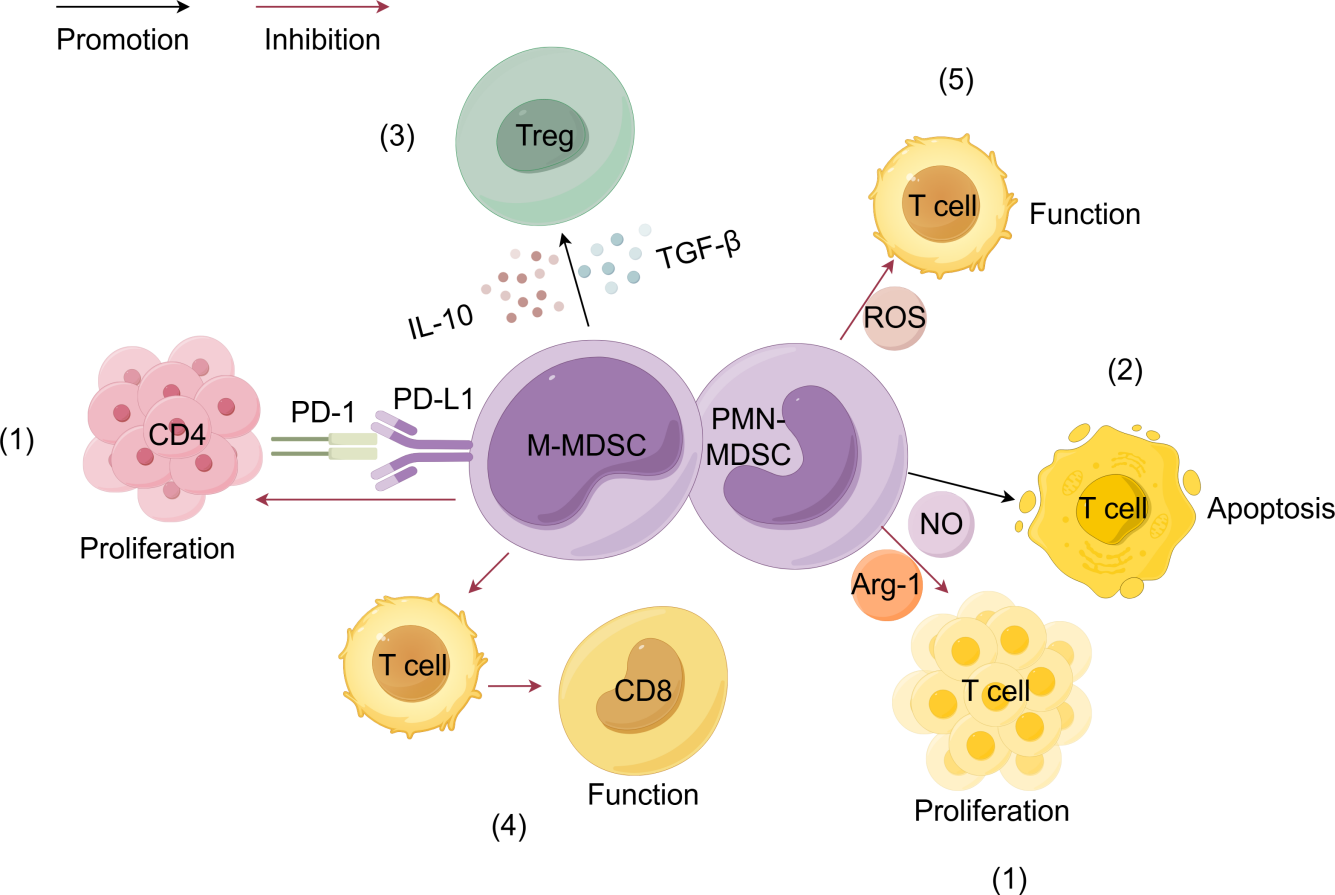
Immunomodulatory mechanisms of MDSCs. (1) MDSCs suppress T cell proliferation by inducing Arg-1 expression and binding PD-L1 to PD-1 on T cells. (2) MDSCs inhibit T cell activity and proliferation while promoting T cell apoptosis by inducing NO production. (3) MDSCs suppress T cell proliferation and function by releasing inhibitory cytokines, including IL-10 and TGF-β. (4) MDSCs inhibit CD8^+^ T cell function by altering T cell activation and migration. (5) MDSCs suppress T cell function by generating reactive oxygen species (ROS).

Clinical investigations have demonstrated that PMN-MDSCs are markedly more prevalent in the peripheral blood of patients with PTB compared to those with LTBI, suggesting a more pivotal role for MDSC subsets in PTB pathogenesis (78). Additionally, the frequency of PMN-MDSCs is significantly elevated in patients with clinically mild PTB relative to those with severe PTB, indicating a potential association between PMN-MDSCs and PTB severity, as well as their possible role in mitigating inflammation-induced lung injury (78). MDSCs exert significant immunosuppressive effects through various mechanisms, thereby influencing the development and outcomes of PTB. A key mechanism among these is the suppression of immune responses through metabolic reprogramming (79, 80). Specifically, MDSCs induce the expression of arginase-1 (Arg-1), leading to the depletion of L-arginine, which in turn results in a deficiency of L-arginine in T cells, thereby inhibiting their proliferation (79). Furthermore, MDSCs can express high levels of inducible nitric oxide synthase (iNOS), catalyzing the production of substantial amounts of nitric oxide (NO). NO acts to suppress T cell activity and proliferation while promoting T cell apoptosis (80). MDSCs secrete cytokines such as IL-10 and TGF-β, which serve to inhibit T cell proliferation and function while promoting Treg expression, thereby contributing to a multifaceted immunosuppressive milieu (81, 82). Empirical evidence suggests that in individuals with PTB and recent Mtb infection, MDSCs attenuate CD4+ T cell function by diminishing cytokine responses and inhibiting CD4+ T cell proliferation, consequently impairing protective T cell responses (83). Furthermore, MDSCs are implicated in the suppression of CD8+ T cell function by modulating T cell activation and migration (83). Research has demonstrated that PD-L1 expressed on the surface of MDSCs post-Mtb infection can interact with PD-1 receptors on T cells, thereby inhibiting T cell activation and proliferation (84). Additionally, MDSCs are capable of producing elevated levels of reactive oxygen species (ROS) through the release of superoxide and various molecular reactions, further suppressing T cell function (85). The immunosuppressive mechanisms employed by MDSCs are illustrated in **Figure 3**.

Acknowledging the immunosuppressive role of MDSCs in PTB, therapeutic strategies targeting these cells demonstrate significant potential. These strategies are designed to mitigate the immunosuppressive effects of MDSCs and bolster the host’s anti-tuberculosis immune response. Current therapeutic interventions targeting MDSCs primarily focus on regulating their proliferation, inducing their differentiation, and inhibiting their immunosuppressive functions (86–88). Research suggests that in murine models, macrophage autophagy can enhance T-cell immune responses by suppressing MDSC production, thereby managing high-dose Mtb infection. This discovery provides a theoretical foundation for modulating MDSC numbers through the induction of autophagy (86). Furthermore, studies indicate that certain tyrosine kinase inhibitors (TKIs) can reduce MDSC levels in cancer patients, thereby enhancing T-cell activity and offering potential insights for PTB treatment (89). In terms of induced differentiation, evidence suggests that treatment with all-trans retinoic acid (ATRA) in TB patients may reduce the inhibitory function of MDSCs by promoting their differentiation into mature myeloid cells (88). The data suggest that post-ATRA administration, the reduction in the frequency of MDSCs is minimal and insufficient to restore T-cell function (88). Conversely, research has demonstrated that in murine models of TB, the combination of ATRA with conventional anti-tuberculosis medications can reduce treatment duration and decrease the likelihood of disease recurrence. Consequently, further investigation into the application of ATRA is warranted (90). Additionally, studies indicate that sildenafil, a phosphodiesterase-5 inhibitor (PDE-5i), augments cyclic guanosine monophosphate (cGMP) levels in MDSCs, leading to decreased expression of Arg-1 and nitric oxide synthase 2 (NOS2), thereby diminishing the immunosuppressive activity of MDSCs (87). In a murine model of MTB infection, sildenafil in combination therapy reduced the bacterial clearance time in the lungs by one month compared to standard chemotherapy. This finding suggests that the integration of sildenafil into conventional anti-tuberculosis treatment regimens may potentially shorten the treatment duration for patients with PTB (91). Alternatively, restoring T cell function may be achieved by directly employing Arg-1 inhibitors (such as Nor-NOHA), iNOS inhibitors (such as 1400W), and using antioxidants to neutralize ROS generated by MDSCs (85, 92, 93). Studies have shown that antibody-mediated antagonism of the LILRB2 receptor can induce MDSCs to adopt an M1 macrophage phenotype, thereby enhancing their ability to kill Mtb (94). This strategy not only helps improve immune control of TB but may also serve as an adjunctive treatment for the disease. In a macaque model, the use of IDO inhibitors has been shown to improve the immune control of tuberculosis, further supporting the potential of MDSCs as therapeutic targets (95).

## Macrophages

Macrophages, as the primary target cells for Mtb infection, play a pivotal role in the development and progression of TB due to their inherent heterogeneity and plasticity (96). During Mtb infection, macrophages can undergo polarization into two distinct functional subtypes, M1 and M2, each characterized by unique immune responses (97). M1 macrophages are associated with pro-inflammatory responses, while M2 macrophages are linked to anti-inflammatory responses and tissue repair. The equilibrium between these two polarization states is essential for the progression of tuberculosis and the regulation of immune responses (98). The M1 macrophages predominantly express the CD68+CD86 phenotype, whereas M2 macrophages primarily express the CD68+CD206 phenotype (99). Alveolar macrophages (AMs) play a crucial role in managing immune responses and ensuring the stability of lung function. Based on cellular origin and surface marker expression, they can be classified into two principal groups: tissue-resident alveolar macrophages (TR-AMs) and monocyte-derived alveolar macrophages (MDMs) (100). Furthermore, a recent study identified a novel macrophage subpopulation, referred to as the grna.2 macrophage subpopulation, within a zebrafish model of tuberculosis (101). Research has confirmed that this subpopulation can suppress excessive inflammatory responses, reduce macrophage pyroptotic death, and promote T cell infiltration, thereby controlling bacterial proliferation (101).

The interactions between macrophages and Mtb are crucial in the pathogenesis of TB. During the initial stages of infection, alveolar macrophages encounter Mtb and recognize it through various pattern recognition receptors (PRRs), including Toll-like receptors (TLRs), Nod-like receptors (NLRs), and C-type lectin receptors (CLRs) (10). Among these, macrophage galactose-type lectin (MGL), a CLR, is notably upregulated upon Mtb exposure. Elevated levels of MGL have been observed in the granulomas of TB patients (102). Studies have shown that silencing MGL leads to increased Mtb replication within macrophages, underscoring MGL’s critical function in innate immunity (102). Following Mtb recognition, macrophages form phagolysosomes by fusing with lysosomes. The acidification of these phagolysosomes activates mechanisms such as the production of toxic antibacterial effectors and autophagy, which serve to degrade the pathogen (103). Tax1bp1 acts as an autophagy receptor, and recent research suggests that a deficiency in Tax1bp1 significantly influences autophagy, cell death, and inflammatory responses in infected macrophages, while also reducing Mtb proliferation (104). The study further substantiates the critical role of the interaction between Tax1bp1 and galactose-type lectin-8 (MGL-8) in enabling macrophages to effectively target Mtb for selective autophagy. Additionally, it was identified that enhancing the expression of MGL-8 can regulate Mtb replication within macrophages, suggesting a potential therapeutic target for tuberculosis treatment (105). Research indicates that functional heterogeneity among specific subpopulations of alveolar macrophages is particularly influential in determining the outcome of Mtb infection (106). In the advanced stages of Mtb infection, the capacity of CD38+ MDMs and a subset of CD38+ TR-AMs to inhibit bacterial growth progressively increases. Notably, CD38+ TR-AMs display unique chromatin accessibility characteristics even prior to infection, indicating an epigenetically primed state that predisposes them to pro-inflammatory differentiation, thereby enhancing their ability to restrict Mtb proliferation (106). The metabolic state of macrophages is intricately linked to their functional capacity, with metabolic reprogramming significantly influencing macrophage polarization and consequently modulating their role in inflammatory responses (107, 108). Initially, glucocorticoids facilitate the production of anti-inflammatory metabolites by altering mitochondrial metabolism in macrophages, thereby inhibiting the synthesis of pro-inflammatory cytokines (107). Additionally, IL-10 enhances the anti-inflammatory capabilities of macrophages by suppressing glycolysis and promoting oxidative phosphorylation within these cells (108). Furthermore, research suggests that biomaterials targeting mitochondrial function can influence macrophage polarization, providing novel perspectives for the treatment of inflammatory diseases (109). These studies lay the theoretical groundwork for the development of innovative anti-tuberculosis therapies.

As our knowledge of macrophages’ role in TB expands, these cells have become a central point in therapeutic research. Traditional antimicrobial drugs predominantly target bacterial components; however, host-directed therapies (HDTs) are designed to enhance anti-TB immunity by modulating the host’s immune response, representing a novel direction in TB treatment (110, 111). Recent advancements in nanotechnology have further facilitated macrophage-targeted drug delivery (112, 113). In a recent study, Liao et al. developed a macrophage-targeted manganese dioxide nanomaterial, termed Tuf-Rif@HA-MnO_2_ nanoparticles (NPs) (114). In murine models, this approach was shown to not only deliver rifampicin directly to macrophages for the eradication of Mtb but also to interact with intracellular glutathione (GSH) to release Mn²^+^ within Mtb-infected macrophages. This release of Mn²^+^ activates the cGAS-STING signaling pathway and promotes macrophage-mediated autophagy. The resulting synergistic effect enhances the clearance of Mtb more effectively (114). Secondly, numerous studies have corroborated that nanoparticles are capable of achieving controlled drug release and targeted delivery to infected macrophages. This capability not only enhances drug bioavailability but also mitigates toxicity to healthy tissues (115, 116). Additionally, extensive research has demonstrated that modulating the autophagy process and lipid metabolism in macrophages can substantially influence the survival and proliferation of MTB (117, 118). For instance, in murine models, the application of fatty acid β-oxidation (FAO) inhibitors has been shown to effectively enhance the antimicrobial capacity of macrophages, thereby significantly inhibiting the growth of Mtb (119). Furthermore, studies indicate that rifabutin-loaded β-glucan microparticles can induce autophagy in Mtb-infected macrophages, resulting in a significant reduction in Mtb counts, including MDR-TB strains (120). Research has demonstrated that in murine models, the application of SMIP-30, an inhibitor of metal-dependent protein phosphatases (PPMs), induces autophagy in macrophages, consequently reducing Mtb survival within these cells (121). These findings imply that autophagy inducers could serve as effective adjunctive therapies to conventional anti-tuberculosis drugs, providing novel treatment strategies, particularly for patients with MDR-TB and XDR-TB. Furthermore, modulating metabolic pathways in macrophages may enhance their antimicrobial efficacy (122). Additionally, by altering the polarization state of macrophages to favor M1 polarization, their antimicrobial capacity can be further augmented (123, 124).

## Microglia

MTB is not limited to lung infections; its hematogenous spread can affect the central nervous system, leading to tuberculous meningitis (TBM) (125). As resident immune cells of the central nervous system, microglia play a key role in the immune response to TBM (126).

When MTB comes into contact with microglia, it is effectively internalized by the microglia and can replicate to a limited extent within the cells (127). Nevertheless, the capacity of microglia to counteract MTB infection is not without limitations; they also demonstrate a unique dual role in the context of MTB infection within the central nervous system (126, 128). A single-cell transcriptomics study has revealed that complement-activated microglia in cerebrospinal fluid cells and peripheral blood mononuclear cells from children with TBM are associated with persistent meningitis (129). Moreover, microglia release a range of pro-inflammatory cytokines when an infection occurs, which can aid in clearing pathogens or cause neurotoxic effects (128).

Research has found that MTB can induce ferroptosis in microglia via the Sp1-Mettl14-Acsl4 axis; regulating this form of cell death may provide new therapeutic targets for TB (130). Studies suggest that HDTs appears to maintain a reasonable balance between pro-inflammatory and anti-inflammatory responses in TBM and may serve as an adjunct to antibiotic therapy (126). Research in a zebrafish model has revealed that microglia may limit the replication of MTB through autophagy, thereby slowing the progression of the disease to some extent. Activation of this autophagy mechanism may offer new insights for the treatment of TBM (128).

## Interactions among immune regulatory cells

**Figure 4:**
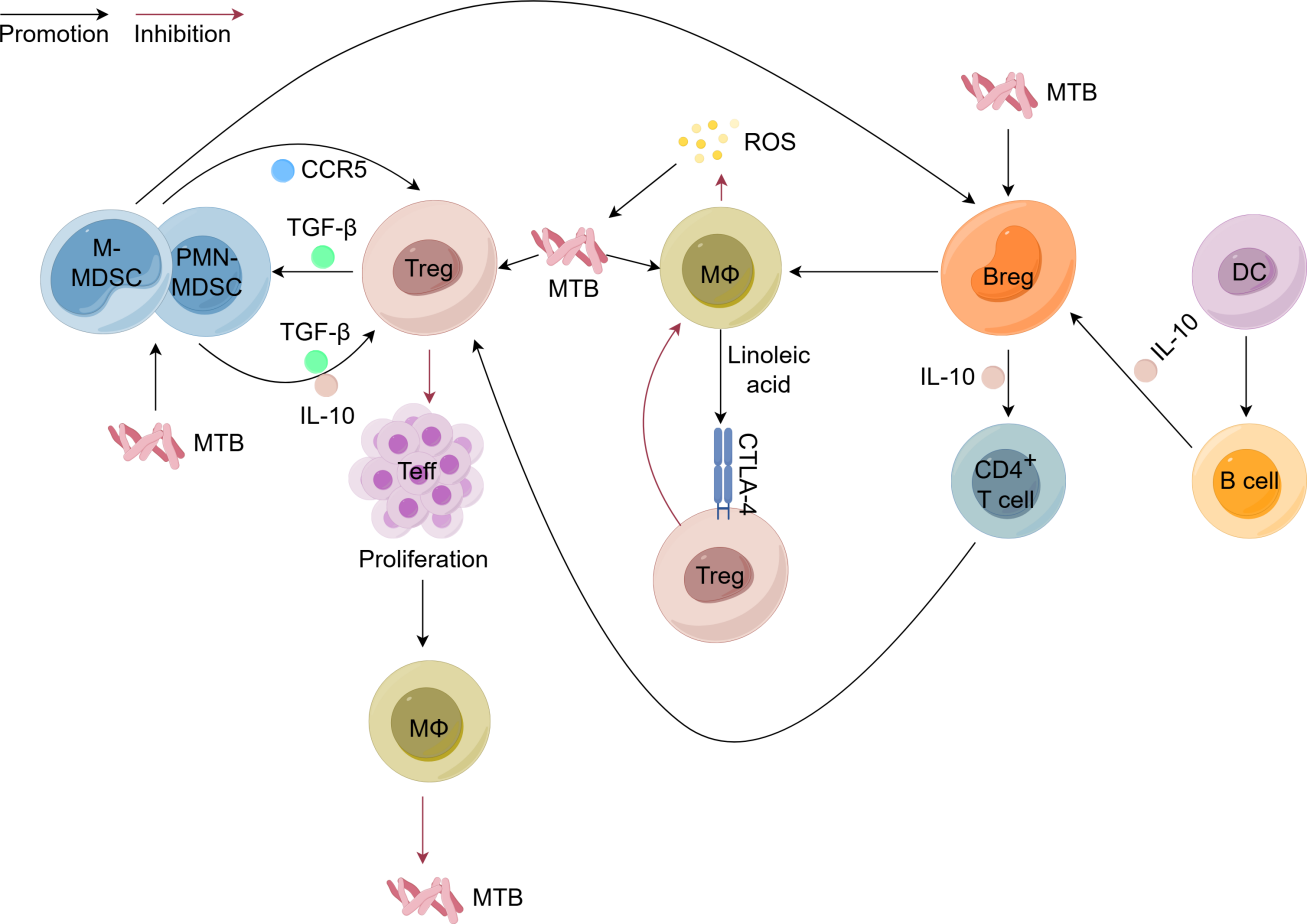
Interactions among various immune regulatory cells in the Mtb infection environment. During the development and progression of tuberculosis, it is regulated by immune regulatory cells such as Tregs, Bregs, MDSCs, and macrophages. These immune cells can interact with each other. On one hand, these immune cells can eliminate Mtb; on the other hand, they may also promote Mtb survival due to excessive immune damage.

Within the immune milieu of PTB, various immunoregulatory cells operate in a highly interconnected manner, engaging in both direct and indirect interactions that influence each other’s functions (131, 132). These cells play dual roles: they can either uphold immune homeostasis or be manipulated by Mtb to aid in its evasion of the host immune response. A deep comprehension of how these regulatory cells interact is essential for creating new immunotherapy approaches. **Figure 4** provides a summary of the interconnections among key immunoregulatory cells, including Tregs, Bregs, MDSCs, and macrophages.

Tregs have been demonstrated to augment the immunosuppressive capabilities of Bregs through direct cell-to-cell interactions (133). Bregs, in turn, facilitate the differentiation of CD4+ T cells into Tregs by secreting cytokines such as IL-10 (56, 62). In patients with PTB, Tregs have been observed to modulate macrophage-mediated control of Mtb by inhibiting the proliferation of effector T cells (134). Furthermore, linoleic acid secreted by Mtb enhances the transport of CTLA-4 in Tregs, thereby suppressing ROS production in macrophages and promoting Mtb survival within these cells (131). Tregs and macrophages may also engage in mutual promotion through additional mechanisms (135). Tregs can further stimulate the expansion of MDSCs by secreting cytokines such as TGF-β (136). Subsets of MDSCs can recruit Tregs by secreting the chemokine CCR5 and interacting with the CXCR6 receptor via the chemokine CXCL16 (136, 137). In the context of lung tumors, MDSC subpopulations have been found to enhance the differentiation and expansion of Tregs (138). In patients with PTB, MDSCs may influence the expansion and function of Tregs through analogous mechanisms, thereby modulating immune responses. Furthermore, Bregs facilitate the polarization of macrophages towards the M2 phenotype and enhance their functional capacity (139). Additionally, subpopulations of MDSCs can promote the generation of Bregs (140). Spleen B cells can be induced by regulatory dendritic cells (DCs) to become IL-10 producing Bregs with a specific phenotype (141). Both macrophages and DCs are capable of modulating the immune response to TB through their interactions with T cells (6, 142). The immunosuppressive network is dynamic, exhibiting significant stage-specificity throughout the progression of the infection. During the initial stages of infection, regulatory dendritic cells and macrophages are the primary cells to encounter Mtb. These cells aim to establish an immunoregulatory dynamic equilibrium by maintaining intracellular antimicrobial control and initiating and sustaining inflammatory responses (6). As Mtb infection advances, the pathogen can enhance its survival and proliferation by altering host cell metabolism and signaling pathways (131). Mtb can trigger inflammasomes by causing stress in the endoplasmic reticulum and damage to mitochondria, leading to the generation of proinflammatory cytokines like IL-1β. This inflammatory response is further amplified through positive feedback mechanisms, leading to an excessive activation of the immune system (143). Additionally, Mtb can impair dendritic cell function, promote T cell polarization towards Th2 and Th17 responses, and suppress Th1 responses, thereby facilitating its persistent infection within the host (144). Furthermore, Mtb induces an increase in regulatory B10 cells by secreting specific lipid molecules, which in turn suppress CD4+ Th1 immune responses and enhance host susceptibility to Mtb infection (56). At this juncture, the regulatory network transitions from safeguarding the host to favoring the pathogen. The latent infection process of Mtb is contingent upon the immune system’s persistent surveillance of Mtb and the maintenance of granulomatous structures. Disruption of this surveillance mechanism allows latent bacteria the opportunity to reactivate. Research suggests that in individuals infected with HIV, immune system imbalances, such as the progressive depletion of CD4+ T cells, may facilitate Mtb reactivation, thereby elevating the risk of tuberculosis onset (145). With advancing age, both the quantity and functionality of MDSCs undergo significant alterations, with MDSCs in older adults demonstrating an enhanced ability to suppress T cell proliferation (146). Additionally, studies have identified an increased proportion of Tregs and altered functionality within the elderly population, which, under specific conditions, may contribute to the reactivation of chronic infections (147, 148).

The interplay between these immune-modulating cells is vital for the initiation and development of PTB. Current research has elucidated key mechanisms involving these cells in various diseases, yet some mechanisms remain unclear in PTB patients. Investigating the interactions among immune-modulating cells further could offer new insights for future PTB prevention and treatment.

## Controversies and context-dependent roles of immunoregulatory cells in PTB

When studying the role of immunoregulatory cells in pulmonary tuberculosis, mouse models serve as crucial tools in the research process, yet their differences from human tuberculosis cannot be overlooked. First, the MDSC phenotype in mouse models differs from that in humans. For example, mouse MDSCs highly express Gr1, whereas human MDSCs highly express CD11b and CD33 (149). Moreover, the immune microenvironment in mouse models differs from that in humans. For example, granuloma formation in mouse TB models differs from that in humans (150). In TB immunology research, although several disease-associated immune pathways have been identified, their translational efficacy remains limited across different species. For example, although the immune pathways in mice share certain similarities with human tuberculosis, these findings have not effectively translated into therapeutic strategies for humans (151). Despite some progress in animal models, directly validating the efficacy of various immunomodulatory strategies in human lungs or granulomas remains challenging. For example, although three-dimensional *in vitro* granuloma models can to some extent reproduce the immune response to tuberculosis, they still fall short of replicating the actual conditions in the human body (152). Accordingly, future research should focus more on validating the efficacy and safety of various immunomodulatory strategies in human subjects.

In PTB research, the vast majority of human studies to date can only reveal correlations between immune regulatory cells and clinical indicators, but struggle to establish causal relationships. Research indicates that the immune response in TB patients is primarily mediated by Th1-type CD4+ T cells, which secrete IFN-γ. However, the specific mechanisms underlying this immune response and its causal relationship with disease progression remain unclear (153). Further research has revealed that the severity of tuberculosis is closely associated with neutrophil abundance and lymphocyte deficiency, while showing no significant correlation with antigen-specific CD4+ T cell responses. This finding further underscores the intricate relationship between immune cells and clinical manifestations (154).

## Current limitations and gaps in understanding immunoregulatory networks in PTB

Despite significant advances in recent years regarding immunoregulatory cells in tuberculosis, it is essential to acknowledge the limitations and knowledge gaps within this field before translating these findings into clinical insights or therapeutic strategies. The heterogeneity of the TB patient population represents one of the primary limitations in current research. Research indicates that the immune system undergoes significant changes as we age (155, 156). Among the elderly population, the sensitivity of interferon-gamma release assays (IGRAs) for tuberculosis is reduced, which may be associated with impaired immune function (155). Further, following BCG vaccination in the elderly, the frequency of memory T cells increased, indicating its capacity to induce non-specific adaptive immune responses (156). These studies indicate that age not only affects the number and function of immune cells but may also influence the immune response following vaccination.

The vast majority of current human studies rely on peripheral blood samples to assess the frequency and function of immune-regulatory cells, a methodological choice that carries certain limitations. Evidence indicates that specific immune cell populations exist within pulmonary granulomas of tuberculosis patients, where they exert significant immunoregulatory effects in the local environment. These characteristics are not readily apparent in peripheral blood (157, 158). Research has revealed that alveolar macrophages exhibit distinct activation states and antimicrobial activities across different pulmonary lesions, which are closely correlated with Mycobacterium tuberculosis burden (159). Furthermore, the expansion and function of resident CD4+ T cells in the lungs are significantly higher than in peripheral blood. These cells secrete cytokines such as IL-17, which contribute to controlling Mycobacterium tuberculosis infection (160). Through these studies, we discovered that the immune response to tuberculosis is primarily concentrated within specific microenvironments in the lungs. These localized immune responses exhibit significant differences compared to the immune status observed in peripheral blood. This finding underscores the importance of focusing on localized immune responses when investigating the immunological mechanisms of tuberculosis and provides new perspectives for developing more effective therapeutic strategies.

To transcend current cognitive limitations and truly comprehend the systemic complexity of immunity requires the systematic application of next-generation technological tools and research designs. In PTB research, single-cell sequencing technology has been employed to analyze peripheral blood and lung samples, revealing the dynamic changes in immune cell subsets during disease progression (161). Using spatial and single-cell RNA sequencing technologies, researchers were able to identify distinct cell populations within the granuloma and discovered that the gene expression of these cell populations varied depending on their location within the granuloma (162). Longitudinal study models play a crucial role in assessing tuberculosis progression and treatment response. By conducting longitudinal measurements of sputum smear results in tuberculosis patients, researchers can predict the risk of adverse outcomes and dynamically forecast survival probabilities (163). Beyond this, the study also identified biomarkers associated with tuberculosis susceptibility by integrating gene expression and clinical data, opening new avenues for predicting the reactivation of latent tuberculosis (164). The fundamental limitation of current understanding primarily stems from the lack of human lung tissue data, meaning that many of our descriptions of this immune regulatory network remain speculative. In the future, we may leverage next-generation tools such as single-cell sequencing, spatial transcriptomics, and longitudinal cohort studies to elevate our understanding of tuberculosis immunity to unprecedented heights.

## Conclusion

This study examines the roles and mechanisms of various immunoregulatory cells in PTB. Evidence indicates that these cells are crucial in mitigating excessive immune-mediated damage, preserving tissue homeostasis, and supporting the prolonged survival of Mtb during chronic infection, which is vital in the context of PTB (14, 165, 166).

The increasing prevalence of MDR-TB presents a formidable challenge to global public health. This rise in MDR-TB cases is attributed to the diminished efficacy of conventional anti-tuberculosis treatment regimens in certain contexts (167). Consequently, comprehensive research into the immunosuppressive mechanisms of immunoregulatory cells in PTB, alongside clinical treatment methodologies, is essential for the development of effective therapeutic strategies (168). Immunoregulatory cells in PTB have been identified as both potential therapeutic targets and diagnostic biomarkers (169). Variations in the frequency and functionality of these cells can be leveraged for early diagnosis, disease assessment, and treatment monitoring (169). Furthermore, immunoregulatory cells hold promise as prognostic indicators; by tracking their dynamic changes, the risk of disease recurrence can be anticipated, thereby informing clinical treatment strategies (170). Additionally, these cells can function as vaccine adjuvants, potentially enhancing vaccine efficacy through the induction of positive synergistic effects (171).

In summary, research on PTB immunoregulatory cells provides crucial insights for deepening our understanding of disease mechanisms and developing novel therapeutic strategies.Future research should aim to better integrate fundamental studies with clinical practice to enhance the use of immunoregulatory cells in treating PTB.
